# Transcriptional Activity of PGC-1**α** and NT-PGC-1**α** Is Differentially Regulated by Twist-1 in Brown Fat Metabolism

**DOI:** 10.1155/2012/320454

**Published:** 2012-10-10

**Authors:** Hee-Jin Jun, Thomas W. Gettys, Ji Suk Chang

**Affiliations:** Laboratory of Nutrient Sensing and Adipocyte Signaling, Pennington Biomedical Research Center, 6400 Perkins Road, Baton Rouge, LA 70808, USA

## Abstract

Brown fat expresses two PGC-1**α** isoforms (PGC-1**α** and NT-PGC-1**α**) and both play a central role in the regulation of cellular energy metabolism and adaptive thermogenesis by interacting with a wide range of transcription factors including PPAR**γ**, PPAR**α**, ERR**α**, and NRF1. PGC-1**α** consists of 797 amino acids, whereas alternative splicing of the *PGC-1* **α** gene produces a shorter protein called NT-PGC-1**α** (aa 1–270). We report in this paper that transcriptional activity of PGC-1**α** and NT-PGC-1**α** is differently affected by the transcriptional regulator, Twist-1. Twist-1 suppresses PGC-1**α** but not NT-PGC-1**α**. The inhibition of PGC-1**α** activity by Twist-1 is mediated by direct interaction through the C-terminal region of PGC-1**α** (aa 353–797). Thus, the absence of the corresponding C-terminal domain in NT-PGC-1**α** allows NT-PGC-1**α** to be free from Twist-1-mediated inhibition. Overexpression of Twist-1 in brown adipocytes suppresses transcription of a subset of PGC-1**α**-target genes involved in mitochondrial fatty acid oxidation and uncoupling (CPT1**β**, UCP1, and ERR**α**). In contrast, NT-PGC-1**α**-mediated induction of these genes is unaffected by Twist-1. These findings show that differences in inhibitory protein-protein interactions of PGC-1**α** and NT-PGC-1**α** with Twist-1 lead to differential regulation of their function by Twist-1.

## 1. Introduction

The transcriptional coactivator PGC-1*α* was first identified as a coactivator of PPAR*γ* in brown adipose tissue and is now known to interact with a broad range of nuclear receptors and transcription factors to regulate mitochondrial biogenesis in most tissues but also control adaptive thermogenesis, fatty acid/glucose metabolism, ROS metabolism, and muscle fiber type switching in a tissue-specific manner [1–7]. The function of PGC-1*α* among tissues is regulated by signaling inputs that increase transcription of *PGC*-1*α* and modulate the transcribed protein through tissue-specific posttranslational modifications [8–14]. This allows PGC-1*α* to function as a key regulator to link nutritional and environmental stimuli to the tissue-specific transcriptional programs.

Alternative splicing of *PGC-1*α** produces an additional transcript that encodes a shorter isoform called NT-PGC-1*α* (aa 1–270) [15]. NT-PGC-1*α* is coexpressed with PGC-1*α* in metabolically active tissues and its expression is coregulated by the nutritional and environmental cues which activate the gene [15–17]. PGC-1*α* is a short-lived nuclear protein containing 797 amino acids. A variety of post-translational modifications enhance the stability and activity of PGC-1*α* by decreasing its targeting to the proteosome. In contrast, NT-PGC-1*α* is relatively stable since it is less effectively targeted to the proteosome due to lack of the C-terminal domain involved in proteosomal targeting [15]. Constitutive activation of target genes by NT-PGC-1*α* is effectively limited by a mechanism that sequesters NT-PGC-1*α* to the cytoplasm in a CRM1-dependent manner [16]. NT-PGC-1*α* activity is primarily modulated by increased translocation to the nucleus. PKA-dependent phosphorylation of NT-PGC-1*α* increases its nuclear retention and subsequent recruitment to the transcriptional complexes [16]. 

Another layer of regulation of PGC-1*α* function is mediated by direct interaction with other regulatory proteins. Previous studies have shown that p160^MBP^, RIP140, and Twist-1 bind to PGC-1*α* and repress its transcriptional activity. p160 myb binding protein was originally identified as a protein that interacts with the regulatory domain of PGC-1*α* (aa 200–400) in C2C12 myoblasts [18]. The docking of p160^MBP^ on PGC-1*α* inhibits transcription of PGC-1*α* target genes [9, 18]. RIP140 is a transcriptional corepressor for a number of nuclear receptors in adipose tissue and skeletal muscle where it represses many PGC-1*α* target genes [19]. Mechanistically, RIP140 directly interacts with PGC-1*α* (aa 184–797) and suppresses its activity [20]. Recently, the transcription factor Twist-1 was also identified as a negative regulator of PGC-1*α* in brown adipose tissue. Twist-1 is a helix-loop-helix (HLH)-containing transcription factor involved in early development, apoptosis, cancer, and osteoblast differentiation [21–23]. A recent study reported that Twist-1 is recruited to the PGC-1*α* target genes by docking to the C-terminal domain of PGC-1*α* (aa 350–797) to negatively modulate oxidative metabolism and UCP1-dependent uncoupling in brown adipose tissue [24]. Interestingly, all of these negative regulators bind to the central to C-terminal region of PGC-1*α*, suggesting that these regulators would have little or no inhibitory effect on NT-PGC-1*α* function in the nucleus.

The present study was designed to investigate the effect of known PGC-1*α* repressors on NT-PGC-1*α* function and found that Twist-1 plays a differential role in the regulation of PGC-1*α* and NT-PGC-1*α* activity in brown adipocytes. Twist-1 significantly suppressed PGC-1*α*-mediated activation of CPT1*β*, UCP1, and ERR*α* by docking to the C-terminal region of PGC-1*α*. In contrast, NT-PGC-1*α*-dependent induction of these genes was not affected by Twist-1 due to lack of interaction with Twist-1.

## 2. Materials and Methods

### 2.1. Cell Cultures and Brown Adipocyte Differentiation

COS-1 cells were grown in DMEM supplemented with 10% FBS and 1% penicillin/streptomycin (Invitrogen). Immortalized PGC-1*α*-deficient mouse brown preadipocyte cell lines expressing empty vector, PGC-1*α*, or NT-PGC-1*α* [16] were maintained in DMEM supplemented with 10% FBS, 1% glutamine, and 1% penicillin/streptomycin. Preadipocytes were grown to confluence in culture medium supplemented with 20 nM insulin and 1 nM T3 (differentiation medium). Differentiation of brown adipocytes was induced (day 1) by incubating the cells in differentiation medium supplemented with 0.5 mM isobutylmethylxanthine (IBMX), 0.5 *μ*M Dexamethasone, and 0.125 mM indomethacin for 48 hours. Thereafter, the cells were maintained in differentiation medium until day 7, followed by treatment with dibutyryl cAMP for 4 h.

### 2.2. Luciferase Reporter Assay

COS-1 cells were transiently transfected using Fugene6 (Roche) with following plasmids. For a transcriptional repression assay with RIP140, GAL4-responsive luciferase reporter (pGK), GAL4-DBD-fused mouse ERR*α*-LBD, and plasmids expressing PGC-1*α*-HA, NT-PGC-1*α*-HA, and RIP140 were used. For a transcriptional repression assay with Twist-1, (PPRE)_3_-TK-luc, pSV sport-PPAR*γ*1, and plasmids expressing PGC-1*α*-HA, NT-PGC-1*α*-HA, and Twist-1 were used. pRL-SV40 control plasmid expressing *Renilla* luciferase was used for normalization. Cells were harvested for luciferase assay 48 h after transfection, and luciferase activity was determined using a Promega Dual Luciferase assay kit (Promega). The firefly luciferase activity was normalized with *Renilla* luciferase activity. Data represent mean ± SEM of at least four independent experiments.

### 2.3. Immunoprecipitation and Western Blot

COS-1 cells were transfected with pcDNA3.1-Flag-Twist-1 and pcDNA3.1-PGC-1*α*-HA or pcDNA3.1-NT-PGC-1*α*-HA. Cells were harvested 48 h after transfection, washed with ice-cold PBS, and lysed in IP buffer (20 mM HEPES-NaOH, pH 7.0, 150 mM NaCl, 0.2% NP-40) supplemented with a protease inhibitor cocktail (Roche). Lysates were precleared with protein G-agarose beads and immunoprecipitated with IgG and anti-Flag antibody (Sigma) overnight at 4°C. After washings, immunoprecipitates were subjected to Western blot analysis using anti-PGC-1*α* antibody directed against the N-terminus of PGC-1*α* (aa 1–200) and anti-Flag antibody.

### 2.4. Retroviral Infection

GP-293 cells were cotransfected with pVSV-G and pBABE-zeo or pBABE-zeo-Twist-1 using Profection transfection system (Promega). Following transfection, the cells were incubated at 32°C to increase viral titer. Virus-containing medium was collected, filtered through the 0.45 *μ*m filter, and used to infect target cells. Immortalized PGC-1*α-*deficient mouse brown preadipocyte cells that ectopically express empty vector, PGC-1*α*, or NT-PGC-1*α* [16] were infected with the viral supernatant supplemented with 8 *μ*g/mL polybrene. The medium was aspirated after 2 h and replaced with fresh viral supernatant, and the procedure was repeated. After 8 h of infection, the cells were replaced with fresh DMEM medium supplemented with 10% FBS, 1% glutamine, and 1% penicillin/streptomycin. Selection was initiated with zeocin (Invitrogen) 48 h after infection. 

### 2.5. Real-Time PCR Analysis

Total RNA was isolated from brown adipocytes using Tri-Reagent (Molecular Research Center) and RNeasy kits (Qiagen). For quantitative RT-PCR analysis, 2 *μ*g of RNA samples were reverse transcribed using oligo dT primers and M-MLV reverse transcriptase (Promega), and 4 ng of cDNA were used in quantitative PCR reactions in the presence of a fluorescent dye (Cybergreen, Takara) on Applied Biosystems 7900 (Applied Biosystems). Relative abundance of mRNA was normalized to that of cyclophilin mRNA. The primers for Twist-1, UCP1, CPT1*β*, Cox7a1, ERR*α*, VLCAD, PPAR*α*, and cyclophilin were previously described [15, 16, 24]. 

## 3. Results 

### 3.1. Twist-1 Negatively Regulates PGC-1*α* but Not NT-PGC-1*α*

**Figure 1:**
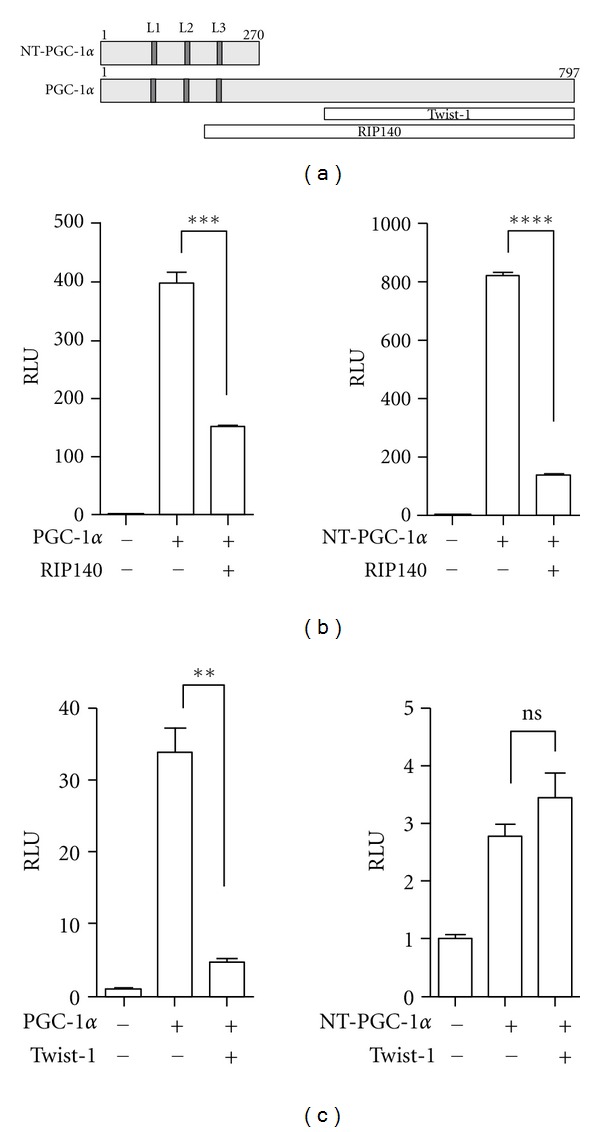
Effects of transcriptional regulators on PGC-1*α* and NT-PGC-1*α* function. (a) Schematic diagram of PGC-1*α* and NT-PGC-1*α* proteins. L1, L2, and L3 represent leucine-rich motifs, one of which (L2) bears the conserved LXXLL sequence that interacts with nuclear receptors. Two white boxes represent the interaction regions of PGC-1*α* with transcriptional regulators, Twist-1, and RIP140, respectively. (b), (c) Transcriptional repression assay using a luciferase reporter. pcDNA3.1, PGC-1*α*, and NT-PGC-1*α* were cotransfected in COS-1 cells with Gal4-ERR*α*-LBD and RIP140 (b) or PPAR*γ* and Twist-1 (c). Luciferase activity was determined 48 h after transfection and the relative luciferase units were calculated as described in Materials and Methods. Data represent mean ± SEM of at least three independent experiments. Significant difference is determined by student *t*-test (**P < 0.01; ***P < 0.001; ****P < 0.0001; ns: not significant).

Brown adipose tissue expresses two PGC-1*α* isoforms (PGC-1*α* and NT-PGC-1*α*), both of which regulate transcription of mitochondrial and thermogenic genes by promoting the activity of several nuclear receptors including PPARs [15–17]. RIP140 and Twist-1, which have been shown to negatively regulate PGC-1*α* activity in adipose tissue, were tested for their effect on regulation of NT-PGC-1*α* function. NT-PGC-1*α* (aa 1–270) partially contains a docking region for RIP140 that binds to the wide region of PGC-1*α* (aa 186–797), whereas the C-terminal Twist-1-binding domain (aa 353–797) is missing in NT-PGC-1*α* (Figure 1(a)). To assess the ability of these transcriptional regulators to modulate NT-PGC-1*α* activity, we carried out transient co-transfection and luciferase reporter assays as described in Materials and Methods. For a transcriptional repression assay with RIP140, Gal4-DBD-fused ERR*α*-LBD was used since RIP140 in part decreases transcriptional activity of full length ERR*α* [25]. The transcriptional activity of Gal4-ERR*α*-LBD was not affected by RIP140 (data not shown). Co-expression of RIP140 with PGC-1*α* significantly inhibited the ability of PGC-1*α* to increase Gal4-ERR*α*-LBD-mediated transcription of the reporter gene (Figure 1(b)). Similarly, RIP140 suppressed NT-PGC-1*α*-mediated induction of the reporter gene (Figure 1(b)), indicating that amino acids 186–270 in NT-PGC-1*α* are sufficient for RIP140 binding and repression. A transcriptional repression assay with Twist-1 showed that Twist-1 largely suppressed the ability of PGC-1*α* to increase PPAR*γ*-mediated transcription, whereas NT-PGC-1*α*-dependent increase of reporter gene expression was not affected by Twist-1 (Figure 1(c)).

### 3.2. Twist-1 Does Not Interact with NT-PGC-1*α*

**Figure 2:**
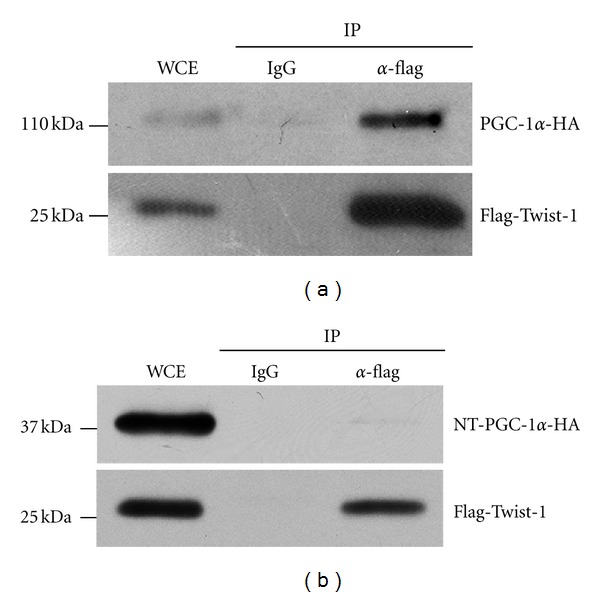
Interactions of Twist-1 with PGC-1*α* but not with NT-PGC-1*α*. PGC-1*α*-HA (a) and NT-PGC-1*α*-HA (b) were co-expressed with Flag-Twist-1 in COS-1 cells. Immunoprecipitates with IgG or anti-Flag antibody were separated by SDS-PAGE and immunoblotted with anti- PGC-1*α* (a, top panel) or HA antibody (b, top panel). Expression of Flag-Twist-1 was confirmed by probing with anti-Flag antibody (a and b, bottom panels).

Twist-1 suppresses PGC-1*α* activity by docking to the C-terminal domain of PGC-1*α* (Figure 1) [24]. To test that no repression of NT-PGC-1*α* activity by Twist-1 is due to lack of interaction between two proteins, Flag-Twist-1 was co-expressed with PGC-1*α*-HA or NT-PGC-1*α*-HA in COS-1 cells and immunoprecipitated with IgG and anti-Flag antibody. PGC-1*α* was efficiently coprecipitated with Twist-1 but not with IgG control (Figure 2(a)). In contrast, NT-PGC-1*α* was not coimmunoprecipitated with Twist-1 (Figure 2(b)), suggesting that Twist-1 is not recruited to NT- PGC-1*α* target genes.

### 3.3. Twist-1 Differentially Regulates a Subset of PGC-1*α*- and NT-PGC-1*α*-Target Genes in Brown Adipocytes

**Figure 3:**
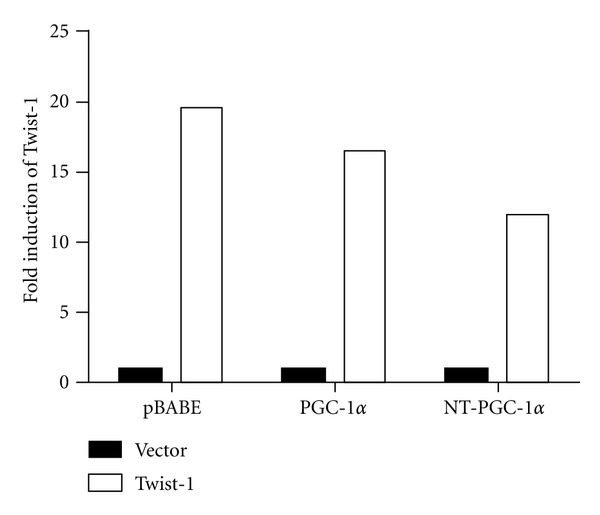
The elevated Twist-1 levels in brown preadipocytes by retroviral infection. Immortalized PGC-1*α-*deficient brown preadipocyte cells that express pBABE-neo vector, PGC-1*α*, or NT-PGC-1*α* were transduced with pBABE-zeo or Twist-1 retrovirus. Zeocine-resistant cells were pooled and Twist-1 expression was assessed by real time-PCR analysis. Relative abundance of Twist-1 mRNA was normalized to that of cyclophilin mRNA.

Twist-1 is selectively expressed in adipose tissue and its overexpression in brown adipocytes specifically suppresses PGC-1*α*-mediated activation of fatty acid oxidation and uncoupling genes [24]. Since NT-PGC-1*α* is co-expressed with PGC-1*α* in brown adipocytes and regulates many PGC-1*α* target genes [15–17], we hypothesized that PGC-1*α* and NT-PGC-1*α* are differently regulated by Twist-1 in brown adipocytes. To investigate a differential role of Twist-1 in PGC-1*α*- and NT-PGC-1*α*-target gene expression in brown adipocytes, the *PGC-1*α**-deficient mouse brown preadipocyte cell lines expressing empty vector, PGC-1*α*, or NT-PGC-1*α* [16] were transduced with Twist-1 retrovirus to overexpress Twist-1. PPAR expression is not affected by PGC-1*α* or NT-PGC-1*α* [26]. In addition, Twist-1 does not change PPAR expression or activity [24]. After retroviral infection, the mRNA levels of Twist-1 were ~16- and ~12-fold increased in the *PGC-1*α**-deficient brown preadipocytes expressing PGC-1*α* and NT-PGC-1*α*, respectively (Figure 3).

**Figure 4:**
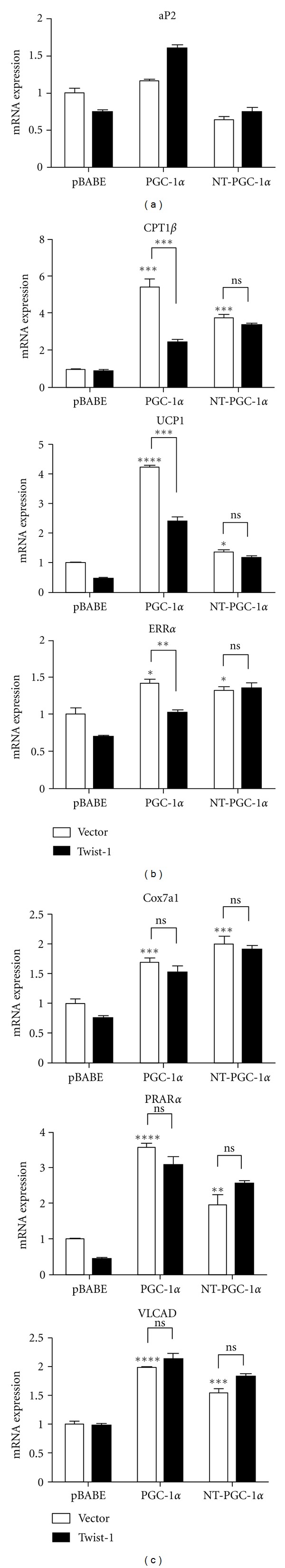
Differential effect of Twist-1 on PGC-1*α* and NT-PGC-1*α* target gene expression in brown adipocytes. (a), (b), (c) Quantitative real time PCR analysis of gene expression in differentiated PGC-1*α-*null brown adipocytes expressing empty vector (pBABE), PGC-1*α*, and NT-PGC-1*α* with empty vector (white bars) or Twist-1 (black bars). Relative abundance of mRNA levels was normalized to that of cyclophilin mRNA. Data represent mean ± SEM of at least four independent experiments. *P < 0.05; **P < 0.01; ***P < 0.001; ****P < 0.0001; ns: not significant.

These brown preadipocyte cell lines were then differentiated for 7 days and treated with dibutyryl cAMP to maximize protein stability/activity of PGC-1*α* and increase nuclear retention of NT-PGC-1*α* [15, 16]. In response to cAMP-induced signaling, p38 MAPK increases stabilization and activation of PGC-1*α* protein by preventing its proteosomal targeting [8], whereas cAMP-activated PKA phosphorylates NT-PGC-1*α*, leading to inhibition of CRM1-mediated nuclear export [16]. Expression of aP2, a marker of adipocyte differentiation, was relatively comparable among the cell lines (Figure 4(a)). In agreement with previous findings [24], overexpression of Twist-1 in differentiated brown adipocytes significantly suppressed PGC-1*α*-mediated induction of CPT1*β*, UCP1, and ERR*α*, which are implicated in mitochondrial fatty acid oxidation and uncoupling (Figure 4(b)). In contrast, Twist-1 had no suppressive effect on NT-PGC-1*α*-dependent induction of CPT1*β*, UCP1, and ERR*α* (Figure 4(b)), indicating that Twist-1 differently regulates PGC-1*α* and NT-PGC-1*α* activity in brown adipocytes. With no addition of dibutyryl cAMP, PGC-1*α* and NT-PGC-1*α* are able to activate their target gene expression although their transcriptional activity is reduced. In the absence of cAMP signaling activation, cAMP-dependent increase of basal UCP1 levels was ~80% reduced, leading to large fold changes in PGC-1*α*- and NT-PGC-1*α*-mediated induction of UCP1 (12.2-fold and 10.3-fold, respectively) (Figure S1, available online at doi: 10.1155/2012/320454). Similarly, Twist-1 significantly suppressed PGC-1*α*-mediated induction of UCP1, whereas NT-PGC-1*α*-mediated increase of UCP1 gene expression was not affected by Twist-1 (Figure S1, available online at doi: 10.1155/2012/320454). Expression of many mitochondrial genes and nuclear receptors is also regulated by PGC-1*α* and NT-PGC-1*α* [15–17]. However, neither PGC-1*α*- nor NT-PGC-1*α*-dependent induction of Cox7a1, PPAR*α*, and VLCAD (Figure 4(c)) and MCAD, Atp5b (not shown) was affected by Twist-1. This suggests that Twist-1 negatively regulates only a subset of PGC-1*α* target genes in brown adipocytes.

## 4. Discussion

We previously reported that PGC-1*α* and NT-PGC-1*α* regulate a number of mitochondrial and thermogenic genes in brown adipose tissue [15–17]. Sympathetic stimulation of BAT by cold increases transcription of the *PGC-1*α** gene by activating and recruiting cAMP-dependent transcription factors, ATF2 and CREB, to the *PGC-1*α** promoter [1, 27]. Subsequent normal and alternative splicings of the transcribed RNA produce comparable mRNA levels of PGC-1*α* and NT-PGC-1*α*, respectively [15]. However, two transcripts produce structurally different proteins that possess fundamental differences in their protein size, stability, and localization. These different natures of two PGC-1*α* isoforms require different regulatory mechanisms to increase their transcriptional activity in response to the same signaling inputs. For example, cold/cAMP-activated p38 MAPK phosphorylation leads to stabilization and activation of PGC-1*α* protein by preventing its proteosomal targeting [8]. In contrast, cAMP-activated PKA phosphorylation increases nuclear accumulation of NT-PGC-1*α* and subsequent recruitment to the transcriptional complexes [16]. 

Here we show an additional regulatory mechanism that differently modulates transcriptional activity of PGC-1*α* and NT-PGC-1*α* in the nucleus of brown adipocytes. The mode of action is mediated by direct interaction of PGC-1*α* with a negative regulator Twist-1, which is abundantly expressed in brown adipocytes. Twist-1 is recruited to PGC-1*α* target genes by docking to the C-terminal region of PGC-1*α* and inhibits their expression by subsequently recruiting the histone deacetylase HDAC5 to the PGC-1*α* target gene promoters (e.g., UCP1 and CPT1) [24]. In contrast, Twist-1 has no inhibitory effect on NT-PGC-1*α*-mediated induction of NT-PGC-1*α* target genes since NT-PGC-1*α* does not recruit Twist-1 to its target gene promoters. Despite potential inhibition of all PGC-1*α* target genes by Twist-1, Twist-1 suppresses only a subset of PGC-1*α* target genes, including UCP1, CPT1*β*, and ERR*α*. Twist-1 is a basic helix-loop-helix (bHLH)-containing transcription factor that binds to the canonical E-box and the related sequences in the regulatory regions of target genes [28], thus raising a possibility that the presence of potential E-boxes on the PGC-1*α* target gene promoters further specifies a subset of PGC-1*α*/Twist-1 target genes. However, it seems unlikely that subsequent docking of Twist-1 to the potential E-boxes on the PGC-1*α* target gene promoters is required for its inhibitory effect since Twist-1-mediated suppression does not depend on its DNA-binding activity [24]. Instead, Twist-1 exerts its transcriptional repression on PGC-1*α* target genes by altering chromatin conformational states by recruitment of histone deacetylases (HDAC) [24]. Thus, it is likely that subsequent recruitment of additional regulators (e.g., histone deaceylases) to the PGC-1*α* target gene promoters by Twist-1 is required for its suppression of PGC-1*α*-mediated gene expression. 

## 5. Conclusion

Our findings demonstrate a differential regulation of PGC-1*α* and NT-PGC-1*α* activity by Twist-1 in brown adipocytes. Twist-1 suppresses PGC-1*α*-mediated transcriptional activation of a subset of PGC-1*α* target genes, including UCP1, CPT1*β*, and ERR*α*. In contrast, NT-PGC-1*α*-mediated induction of these genes is not affected by Twist-1.

## Supplementary Material

Figure S1. Differential effect of Twist-1 on PGC-1*α*- and NT-PGC-1*α*-dependent induction of UCP1.
Quantitative real time PCR analysis of gene expression in differentiated PGC-1*α*-null brown adipocytes expressing empty vector (pBABE), PGC-1*α*, and NT-PGC-1*α* in the absence (white bars) or presence of Twist-1 (black bars). Differentiated brown adipocytes were collected without treatment with dibutyryl cAMP. Relative abundance of mRNA levels was normalized to that of cyclophilin mRNA. Data represent mean ± SEM of at least four independent experiments. ∗∗∗∗,p < 0.0001; ns, not significant.

## Abbreviations

PGC-1*α*: Peroxisome proliferator-activated receptor *γ* coactivator 1*α*
ERR*α*: Estrogen-related receptor *α*
NRF1: Nuclear respiratory factor 1
CPT1*β*: Carnitine palmitoyltransferase 1*β*
UCP1: Uncoupling proteins 1
ROS: Reactive oxygen species
PKA: Protein kinase A
p160^MBP^: p160 Myb binding protein
RIP140: Receptor-interacting protein 140
MAPK: Mitogen-activated protein kinase
VLCAD: Very long-chain acyl-CoA dehydrogenase
MCAD: Medium-chain acyl-CoA dehydrogenase
ATF2: Activating transcription factor 2
CREB: cAMP response element-binding.
