# Supplementary figures and images for: Transcriptional Activity of PGC-1α and NT-PGC-1α Is Differentially Regulated by Twist-1 in Brown Fat Metabolism
Source: PPAR Res. 2012 Oct 10;2012:320454. doi: 10.1155/2012/320454 (PMC3474972; doi:10.1155/2012/320454)

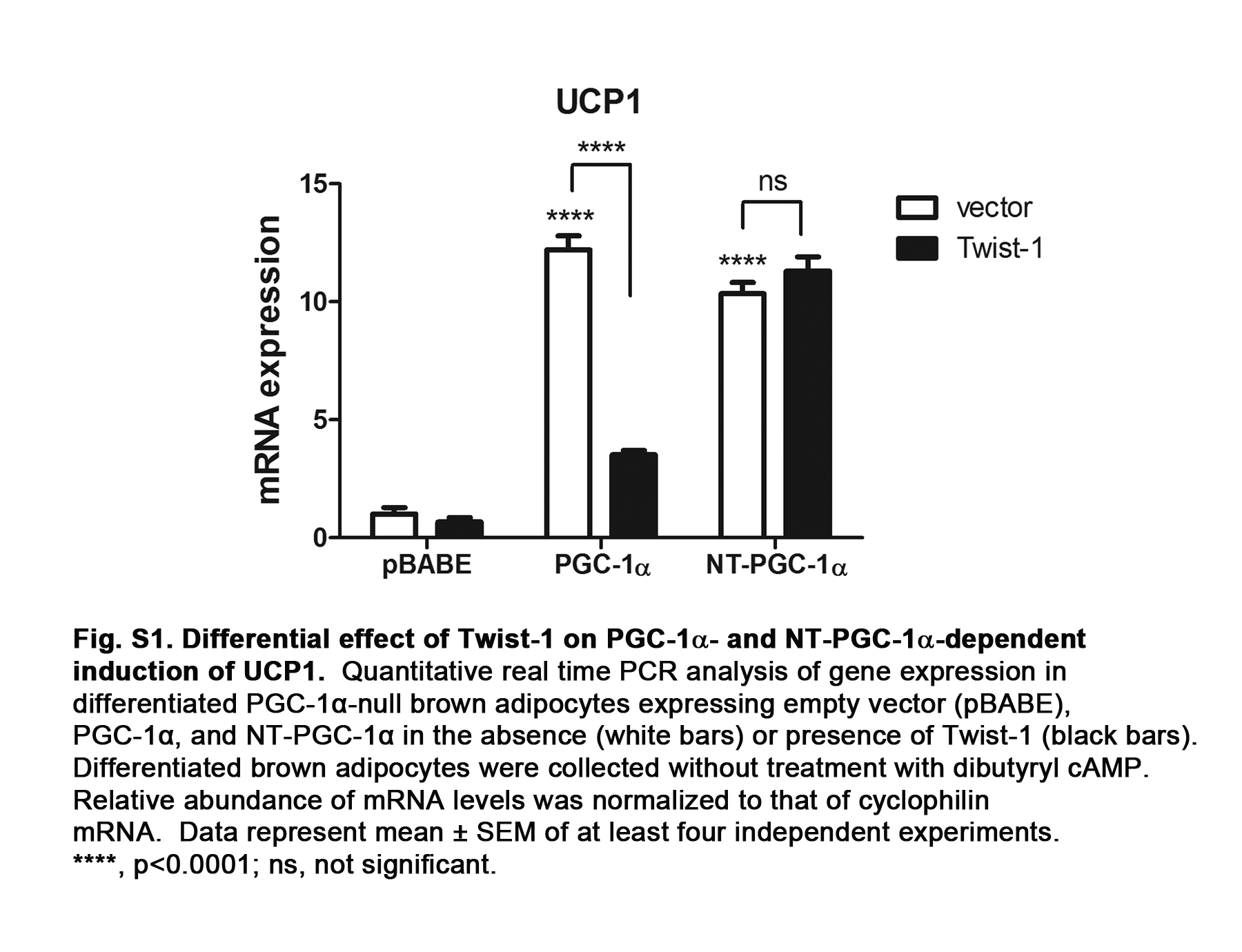

Supplement: Supplementary file 1 — Figure S1. Differential effect of Twist-1 on PGC-1α- and NT-PGC-1α-dependent induction of UCP1. Quantitative real time PCR analysis of gene expression in differentiated PGC-1α-null brown adipocytes expressing empty vector (pBABE), PGC-1α, and NT-PGC-1α in the absence (white bars) or presence of Twist-1 (black bars). Differentiated brown adipocytes were collected without treatment with dibutyryl cAMP. Relative abundance of mRNA levels was normalized to that of cyclophilin mRNA. Data represent mean ± SEM of at least four independent experiments. ∗∗∗∗,p < 0.0001; ns, not significant. [file 320454.f1.tif]
